# Up-Regulation of miR-130b-3p Activates the PTEN/PI3K/AKT/NF-κB Pathway to Defense against *Mycoplasma gallisepticum* (*HS* Strain) Infection of Chicken

**DOI:** 10.3390/ijms19082172

**Published:** 2018-07-25

**Authors:** Bo Yuan, Mengyun Zou, Yabo Zhao, Kang Zhang, Yingfei Sun, Xiuli Peng

**Affiliations:** Key Laboratory of Agricultural Animal Genetics, Breeding and Reproduction Ministry of Education, College of Animal Science and Technology and College of Veterinary Medicine, Huazhong Agricultural University, Wuhan 430070, China; yuanbo_0902@webmail.hzau.edu.cn (B.Y.); zoumengyun@webmail.hzau.edu.cn (M.Z.); zyb@webmail.hzau.edu.cn (Y.Z.); zhangkang123@webmail.hzau.edu.cn (K.Z.); sunyingfei@webmail.hzau.edu.cn (Y.S.)

**Keywords:** *Mycoplasma gallisepticum*, chicken, miR-130b-3p, PTEN/PI3K/AKT/NF-κB pathway

## Abstract

*Mycoplasma gallisepticum* (*MG*) is the pathogen of chronic respiratory disease (CRD), hallmarked by vigorous inflammation in chickens, causing the poultry industry enormous losses. miRNAs have emerged as important regulators of animal diseases. Previous miRNA sequencing data has demonstrated that miR-130b-3p is up-regulated in *MG*-infected chicken embryo lungs. Therefore, we aimed to investigate the function of miR-130b-3p in *MG* infection of chickens. RT-qPCR results confirmed that miR-130b-3p was up-regulated both in *MG*-infected chicken embryo lungs and chicken embryonic fibroblast cells (DF-1 cells). Furthermore, functional studies showed that overexpression of miR-130b-3p promoted *MG*-infected DF-1 cell proliferation and cell cycle, whereas inhibition of miR-130b-3p weakened these cellular processes. Luciferase reporter assay combined with gene expression data supported that phosphatase and tensin homolog deleted on chromosome ten (PTEN) was a direct target of miR-130b-3p. Additionally, overexpression of miR-130b-3p resulted in up-regulations of phosphatidylinositol-3 kinase (PI3K), serine/threonine kinase (AKT), and nuclear factor-κB (NF-κB), whereas inhibition of miR-130b-3p led to the opposite results. Altogether, upon *MG* infection, up-regulation of miR-130b-3p activates the PI3K/AKT/NF-κB pathway, facilitates cell proliferation and cell cycle via down-regulating PTEN. This study helps to understand the mechanism of host response to *MG* infection.

## 1. Introduction

*Mycoplasma* is the simplest and smallest prokaryote widespread in nature, and is distinguished by the absence of a cell wall [1]. It can invade and survive in a wide range of eukaryotes, such as humans, plants, and domestic animals, leading to multiple diseases [2,3,4]. As one of the most important avian mycoplasma pathogens, *Mycoplasma gallisepticum* (*MG*) can cause chronic respiratory disease (CRD) characterized by chronic inflammation in the respiratory tract of chickens [5]. In addition, studies have shown that *MG* is capable of resisting antibiotics, evading host immune system, and then crossing through the respiratory mucosal barrier to establish a systemic infection [6]. On the one hand, *MG* can invade and cohabit with non-phagocytic host cells, such as Hela cells, chicken erythrocytes, and chicken embryo fibroblasts (CEF), and inner organs of chickens in a parasitic way for a long time [6,7]; on the other hand, the majority of surface antigens of *MG* are highly variable [5,8]. Despite great advances in promoting antibiotic and vaccine sensitivity, *MG* infection still occurs frequently in chickens of different ages, especially in the presence of co-infections, bringing great economic losses to poultry industry [9,10,11,12]. Therefore, clarification of the molecular mechanism of *MG* infection is urgently needed. The *MG-HS* strain, used in this study, is a pathogenic strain obtained from a chicken farm in Hubei Province of China [13,14].

miRNAs, a class of short non-coding RNA molecule that is widely distributed in species, are particularly important regulators of gene expression by binding to the untranslated regions of target genes to direct their posttranscriptional repression [15,16]. It is estimated that nearly one third of human and animal genes are regulated by miRNAs, which provides miRNAs the capability to control a wide range of physiological processes, including cell proliferation, cell cycle progression, and inflammatory response [17,18]. Many miRNAs have been reported to play important roles in avian diseases. For instance, in avian Marek’s disease, gga-miR-26 was significantly down-regulated in Marek’s disease virus (MDV)-infected spleens; overexpression of gga-miR-26 suppressed MDV-infected cell proliferation [19]. In avian leukosis, gga-miR-375 was obviously under-expressed in ALV-J infected chicken liver at 20 days post-infection; high expression of gga-miR-375 restrained DF-1 cell proliferation and cell invasion, and promoted cell apoptosis [20].

miR-130b-3p is known to play particularly significant roles in cancer progression in mammals [21,22,23,24,25,26]. Recently, some studies have shown that miR-130b-3p is up-regulated in infectious bursal disease virus (IBDV)-infected DF-1 cells and overexpression of miR-130b-3p could promote beta interferon mRNA level by directly targeting suppressors of cytokine signaling 5 in DF-1 cells and restrained IBDV replication via targeting the IBDV genome [27]. In addition, miR-130b-3p has been reported to exert critical roles in various inflammatory diseases [28,29,30,31]. For instance, overexpression of miR-130b could alleviate LPS-induced vascular inflammation by inhibiting interleukin (IL)-6 and (tumor necrosis factor α) TNF-α expression through targeting tumor progression locus 2 [25]. However, the role of miR-130b-3p in *MG* infection has been seldom reported.

Our preliminary deep sequencing data indicated that miR-130b-3p was up-regulated in *MG*-infected chicken embryo lungs [32]. Consistent with the sequencing data, we verified in this study that miR-130b-3p was notably high-expressed both in vitro and in vivo upon *MG* infection. Furthermore, we found that miR-130b-3p could regulate cell proliferation and cell cycle in host defense against *MG* infection by regulating the PI3K/AKT/NF-κB pathway through directly targeting PTEN.

## 2. Results

### 2.1. Upon MG Infection, miR-130b-3p Was Up-Regulated Both In Vivo and In Vitro

**Figure 1:**
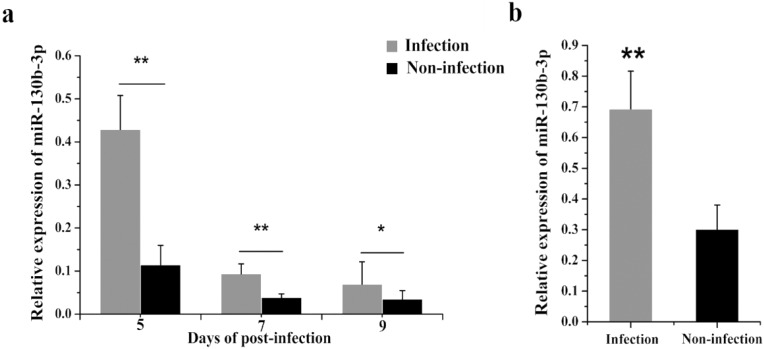
miR-130b-3p was highly expressed in both *MG*-infected chicken embryo lungs and DF-1 cells. (**a**) The relative level of miR-130b-3p in *MG*-infected chicken embryo lungs. Total RNA were extracted from frozen infected chicken embryo lungs on days 5, 7, and 9 post-infection (equivalent to days 14, 16, and 18 of eggs hatching) using TRNzol Universal. Then, the level of miR-130b-3p in *MG* infected embryo chicken lungs was determined through RT-qPCR; (**b**) The relative level of miR-130b-3p in *MG*-infected DF-1 cells. Cells cultivated in 6-well culture dishes were treated with 200 μL *MG* (1 × 10^10^ CCU/mL). After 24 h treatment, total RNA of infected cells were extracted using TRNzol Universal. The level of miR-130b-3p-infected cells was detected by RT-qPCR. The data was normalized to 5S-rRNA. Each experiment group contained at least three duplicates. Each duplicate was measured at least three times. All values are expressed as mean ± SD. Marked differences were expressed as * *p* < 0.05, ** *p* < 0.01.

A previous deep sequencing revealed that miR-130b-3p was overexpressed in *MG*-infected chicken embryo lungs. To confirm the deep sequencing result, we examined miR-130b-3p expression in both *MG*-infected chicken embryo lungs and DF-1 cells. As shown in Figure 1, miR-130b-3p levels were markedly increased in *MG*-infected DF-1 cells (Figure 1b) and chicken embryo lungs on days 5, 7, and 9 post-infection (equivalent to days 14, 16, and 18 of eggs hatching) (Figure 1b), as well, which implied that miR-130b-3p may be involved in *MG* infection.

### 2.2. miR-130b-3p Promoted Proliferation of MG-Infected DF-1 Cells by Accelerating Cell Cycle Progression

**Figure 2:**
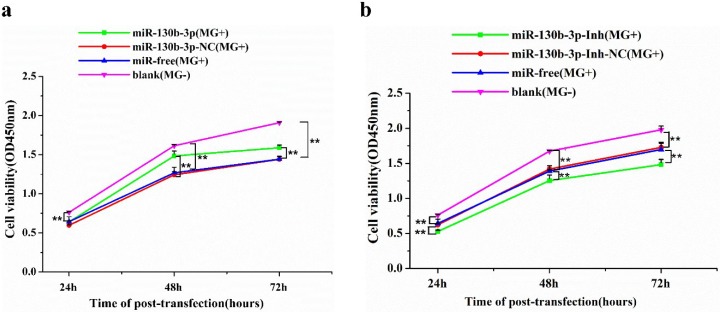
miR-130b-3p promoted cell proliferation of *MG*-infected DF-1 cells. (**a**) Overexpression of miR-130b-3p promoted proliferation of *MG*-infected DF-1 cells; (**b**) Inhibitors of miR-130b-3p restrained proliferation of *MG*-infected DF-1 cells. DF-1 cells cultured in 96-well culture dishes were incubated with 10 μL *MG* (1 × 10^10^ CCU/mL) for 2 h. Then, the infected cells were transfected with miR-130b-3p, miR-130b-3p-NC, miR-130b-3p-Inh or miR-130b-3p-Inh-NC. 24, 48, and 72 h after transfection, respectively, a microplate reader was applied to examine the viability of DF-1 cells using the CCK-8. The absorbance was measured at 450 nm. Values are expressed as mean ± SD (*n* = 6). Marked differences were expressed as ** *p* < 0.01.

Cell proliferation plays a critical role in host defend against microbial infection. Thus, we further investigated whether miR-130b-3p had an effect on DF-1 cells proliferation during *MG* infection by transfecting miR-130b-3p mimics into DF-1 cells. Expectedly, all the *MG*-infected groups, including the miR-130b-3p mimics group, miR-130b-3p-NC group, and miR-free group, showed obvious decreases in cell proliferation compared with the blank *MG*-group during *MG* infection. Interestingly, at 48 h post-transfection, the inhibited cell proliferation was restored by miR-130b-3p mimics (*MG*+) to a certain extent compared with the control groups (Figure 2a). A similar result was obtained at 72 h post-transfection. Then, we assessed the impact of the miR-130b-3p inhibitor on cell proliferation. Expectedly, the highest DF-1 cells growth curve was observed in the blank group (*MG*-) compared with all the *MG*-infected groups during *MG* infection. During 48–72 h post-transfection, we found a dramatic decrease in the cell growth curve of miR-130b-3p-Inh group compared with the miR-130b-3p-Inh-NC (*MG*+) group, the miR-free group (*MG*+) or the blank group (Figure 2b). These results suggest that miR-130b-3p antagonized *MG*-mediated inhibition of cell proliferation.

**Figure 3:**
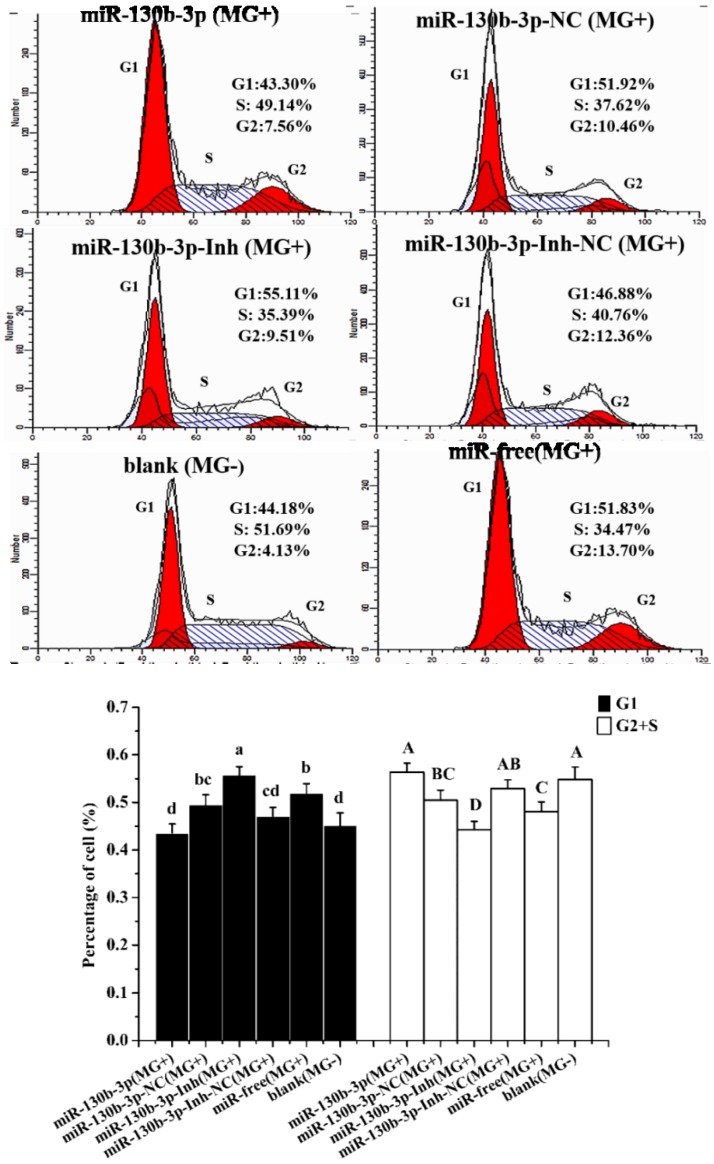
miR-130b-3p facilitated cell cycle progression upon *MG* infection. DF-1 cells cultured in 6-well culture dishes were incubated with 200 μL *MG* (1 × 10^10^ CCU/mL) for 2 h. Then, the infected cells were transfected with miR-130b-3p, miR-130b-3p NC, miR-130b-3p-Inh or miR-130b-3p-Inh-NC. 48 h after transfection, a flow cytometer was applied to analyze the cell phase distribution. Each experiment group contained at least three duplicates. Each duplicate was measured at least three times. Values are expressed as mean ± SD. Bars with different letters in lowercase and uppercase indicate significant difference (*p* < 0.05) in G1 phase and G2/S phase, respectively.

To figure out whether the increased cell count at 48 h post-transfection was attributed to the progress of cell division cycle, we further performed the cell cycle distribution analysis. As demonstrated in Figure 3, *MG* restrained mitosis by blocking 51.83% of the DF-1 cells at G1 phase compared with 44.18% in the blank group. Overexpression of miR-130b-3p significantly improved the total number of DF-1 cells distributed in S and G2 phases, and correspondingly reduced the cell distribution in G1 phase (43.30%), compared with 51.92% in miR-130b-3p-NC (*MG*+) group, opposing *MG*-induced cellular proliferation inhibition. In contrast, low expression of miR-130b-3p arrested the cells at G1 phase (55.11%), compared with 46.88% in miR-130b-3p-Inh-NC (*MG*+) group, and thereby reduced the total number of DF-1 cells distributed in S and G2 phases. Collectively, upon *MG* infection, overexpression of miR-130b-3p is closely implicated in promoting cell proliferation by accelerating cell cycle progression.

### 2.3. PTEN Was a Direct Target of miR-130b-3p in DF-1 Cells

**Figure 4:**
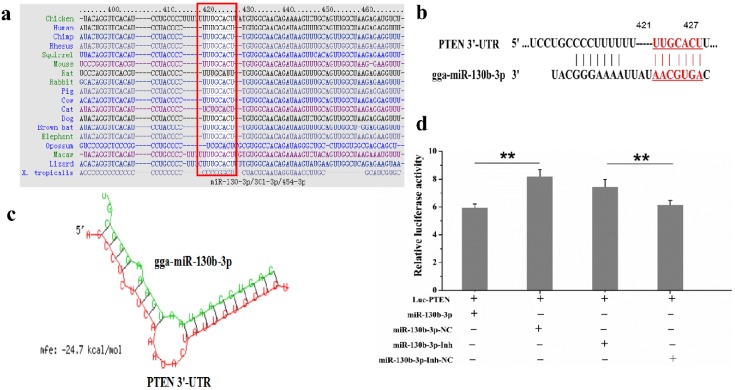
PTEN was a direct target of miR-130b-3p in DF-1 cells. (**a**) Prediction of conservation of the PTEN 3′-UTR in different species by TargetScan. The conserved target sequences are highlighted. (**b**) Prediction of binding site for miR-130b-3p from the PTEN 3′-UTR. The binding domain from position 421 to 427 is highlighted red. (**c**) Prediction of the secondary structure of the RNA duplex between miR-130b-3p and the PTEN 3′-UTR by RNAhybrid. (**d**) miR-130b-p directly targeted PTEN 3′-UTR in vitro. DF-1 cells cultivated in 24-well culture dishes were co-transfected Luc-PTEN (3′-UTR) with miR-130b-3p, miR-130b-3p-NC, miR-130b-3p-Inh, or miR-130b-3p-Inh-NC. 48 h after transfection, the cell lysates in each group were collected to test the relative luciferase activity by a microplate reader. All the Renilla luciferase activities were standardized by firefly luciferase activities. Values are expressed as mean ± SD. Each experiment group contained at least three duplicates. Each duplicate was measured at least three times. Marked differences are expressed as ** *p* < 0.01.

Given that miRNAs usually exert their functions by regulating target genes, we then performed bioinformatics assays to predict the miR-130b-3p target in chicken. PTEN, one of the key regulators of cell functions and inflammatory response, was selected as a candidate target of miR-130b-3p in chicken based on TargetScan, miRDB, and RNAhybrid analysis. According to TargetScan analysis, PTEN 3′-UTR covers a highly conserved binding site for miR-130b-3p from position 421 to 427 bp in different species (Figure 4a, b). RNAhybrid revealed that the minimum free energy (MFE) of the RNA duplex is −24.7 kcal/mol, suggesting a stable combination between miR-130b-3p and its target PTEN (Figure 4c).

To further confirm whether miR-130-3p could directly target PTEN 3′-UTR, we applied a dual-luciferase reporter assay by co-transfecting DF-1 cells with Luc-PTEN 3′-UTR and miR-130b-3p mimics, miR-130b-3p inhibitor, miR-130b-3p-Inh-NC, or miR-130b-3p-NC. As demonstrated in Figure 4d, overexpression of miR-130b-3p obviously decreased the luciferase activity of Luc-PTEN 3′-UTR, whereas the transfection of miR-130b-3p-NC did not impact the luciferase activity. Conversely, the inhibitor of miR-130b-3p significantly increased the activity of the Luc-PTEN 3′-UTR vector. As expected, miR-130b-3p-Inh-NC did not impact the luciferase activity either. Collectively, miR-130b-3p directly targets to PTEN by binding to its 3′-UTR in DF-1 cells.

### 2.4. miR-130b-3p Negatively Regulated PTEN Expression Both In Vivo and In Vitro

**Figure 5:**
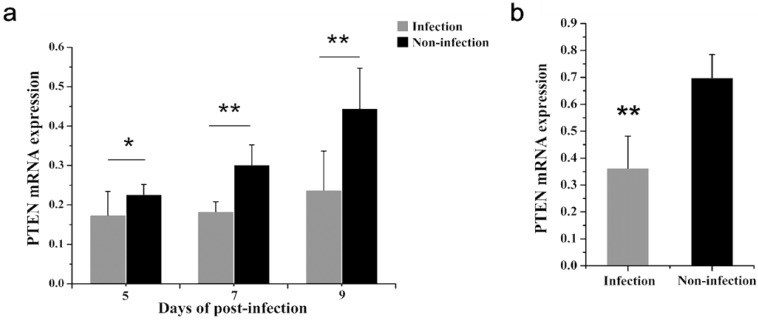
PTEN was down-regulated in both *MG*-infected chicken embryo lung tissues and DF-1 cells. (**a**) The relative mRNA level of PTEN in *MG* infected chicken embryo lungs. Total RNA were extracted using TRNzol Universal from frozen infected chicken embryos lungs on days 5, 7, and 9 post-infection (equivalent to days 14, 16, and 18 of eggs hatching). Then, the relative mRNA level of PTEN in *MG*-infected lungs was detected by RT-qPCR. (**b**) The relative mRNA level of PTEN in *MG*-infected DF-1 cells. DF-1 cells cultured in six-well culture dishes were incubated with 200 μL *MG* (1 × 10^10^ CCU/mL) for 24 h. Then, the cells were harvested. Total RNA of *MG*-infected DF-1 cells were extracted using TRNzol Universal. PTEN expression in infected DF-1 cells were detected by RT-qPCR. GAPDH was served as an internal control. Each experiment group contained at least three duplicates. Each duplicate was measured at least three times. Values are expressed as mean ± SD. Marked differences are expressed as * *p* < 0.05, ** *p* < 0.01.

**Figure 6:**
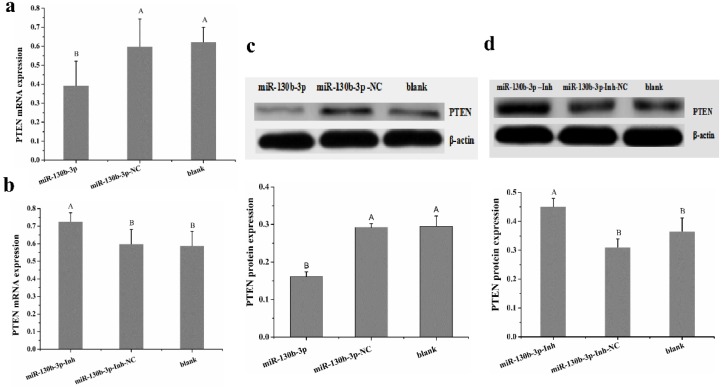
miR-130b-3p inversely regulated PTEN expression. (**a**) Overexpression of miR-130b-3p down-regulated PTEN mRNA expression in DF-1 cells. (**b**) Inhibitor of miR-130b-3p increased PTEN mRNA expression in DF-1 cells. (**c**) Overexpression of miR-130b-3p down-regulated PTEN protein expression in DF-1 cells. (**d**) Inhibitor of miR-130b-3p increased PTEN protein expression in DF-1 cells. Each experiment group contained at least three duplicates. Each duplicate was measured at least three times. Values are expressed as mean ± SD. Bars with different superscripts letters show marked differences (*p* < 0.01).

We next evaluated whether PTEN is involved in *MG* infection. In contrast to the expression pattern of miR-130b-3p (Figure 1a,b), we detected a lower expression level of PTEN in *MG*-infected DF-1 cells (Figure 5b) and chicken embryo lungs on days 5, 7, and 9 post-infection (equivalent to days 14, 16, and 18 of eggs hatching) (Figure 5a) compared with the corresponding control group. Based on the above results, we further determine whether the down-regulation of PTEN was attributable to high expression of miR-130b-3p upon *MG* infection. At 48 h post-transfection, the cells overexpressing miR-130b-3p had markedly decreased PTEN mRNA expression, as well as protein expression (Figure 6a,c). Conversely, the cells under-expressing miR-130b-3p had remarkably enhanced PTEN mRNA expression, as well as protein expression (Figure 6b,d). Collectively, miR-130b-3p negatively regulated endogenous PTEN by targeting its 3′-UTR.

### 2.5. miR-130b-3p Activated the PI3K/AKT/NF-κB Pathway.

**Figure 7:**
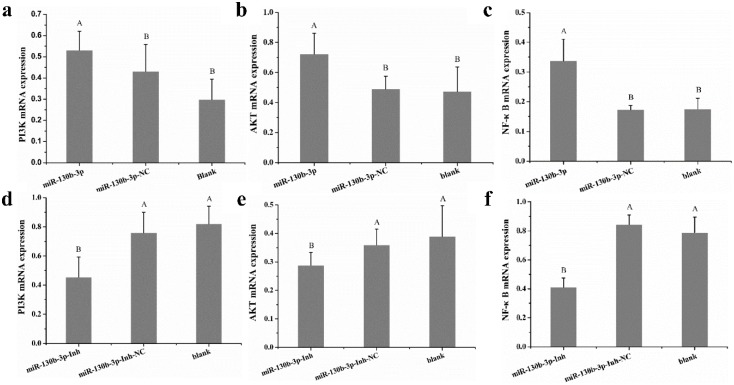
miR-130b-3p activates PI3K/AKT/NF-κB pathway. DF-1 cells cultured in six-well culture dishes were transfected with miR-130b-3p or miR-130b-3p-NC. After 48 h, the expressions of PI3K (**a**), AKT (**b**), and NF-κB (**c**) were examined by RT-qPCR. Similarly, DF-1 cells transfected with miR-130b-3p-Inh or miR-130b-3p-Inh-NC were incubated for 48 h. Then, the expressions of PI3K (**d**), AKT (**e**), and NF-κB (**f**) were examined through RT-qPCR. The relative expressions of PI3K, AKT, and NF-κB were normalized to GAPDH. Each experiment group contained at least three duplicates. Each duplicate was measured at least three times. Values are expressed as mean ± SD. Bars with different superscripts letters show marked differences (*p* < 0.01).

We further analyzed the functions of miR-130b-3p by its target PTEN in *MG* infection. It has been well reported that the PI3K/AKT signaling pathway is one of the central pathways by which PTEN exerts regulatory roles [33]. In addition, AmiGo (http://amigo.geneontology.org) analysis revealed that PTEN exerts a significant regulatory role in regulating inflammatory pathway, such as NF-κB-mediated signaling pathway and PI3K/AKT pathway. We then evaluated the effects of miR-130b-3p on PI3K, AKT, and NF-κB expressions in DF-1 cells. As shown in Figure 7, up-regulation of miR-130b-3p dramatically elevated PI3K, AKT, and NF-κB expressions compared with control groups (Figure 7a–c). However, the opposite results were obtained when miR-130b-3p expression was inhibited (Figure 7d–f). Altogether, up-regulation of miR-130b-3p activates the PI3K/AKT/NF-κB pathway by down-regulating PTEN in DF-1 cells.

## 3. Discussion

The CRD caused by *MG* is a worldwide avian epidemic, causing great financial losses in the poultry industry. However, no definite systematic therapeutic strategy for *MG* exists. miRNAs are emerging as novel targets for molecular diagnosis and treatment due to their significant effects on gene and signaling pathway regulation. The importance of miRNAs in a wide range of animal diseases has been well documented. However, the functions of miRNAs in *MG*-induced CRD have seldom been reported. On the basis of previous miRNA profile sequencing data, we found that *MG* can modulate the expression of miRNAs in chicken embryo lungs. Subsequently, we confirmed the key roles of gga-miR-101-3p [34], gga-miR-19a [35], gga-miR-99a [36], and gga-miR-451 [37] in host response to *MG* infection, suggesting that miRNAs play important regulatory roles in CRD progression, and a more comprehensive research of differential expressed miRNAs associated with *MG* infection might provide novel therapeutic strategies for CRD.

miR-130b-3p has been reported to regulate inflammatory responses, cell functions, and gene expressions in response to many diseases in mammals [25,31,38]. In a previous miRNA profile analysis, we found that miR-130b-3p was up-regulated in *MG*-infected chicken embryo lungs [32]. Consistent with the sequencing data, we confirmed here that *MG* induced increase of miR-130b-3p expression in both *MG*-infected DF-1 cells (Figure 1b) and chicken embryo lungs on days of 5, 7, and 9 post-infection (Figure 1a). On the basis of this finding, it is reasonable to believe that miR-130b-3p is involved in the host response to *MG* infection.

Owing to their small genome and absence of a cell wall, *mycoplasmas* have very limited biosynthetic capabilities and must rely on the adhesion to host cells for infection and nutrients exchange [1]. During the infection, *mycoplasmas* can deliver mycoplasma components that may damage DNA of host cells, thereby affect cell functions, such as cell proliferation and cell cycle [39]. In addition, it was reported that *mycoplasma* infection had an inhibitory effect on the expression of p53, which is essential for the cell cycle progression [40]. Since the cohabitation of parasites in host requires inhibition of cell apoptosis, we investigated the effect of *MG* on cell proliferation and cell cycle progression. Our results showed that *MG* inhibited DF-1 cell proliferation and blocked cells at G1 phase during the infection (Figure 2 and Figure 3). However, up-regulation of miR-130b-3p enhanced *MG*-infected DF-1 cell proliferation (Figure 2a) and facilitated cell cycle progression (Figure 3), antagonizing *MG*-induced cell damage. In contrast, inhibition of miR-130b-3p resulted in opposite results, which were similar to that of the *MG* group (Figure 2b and Figure 3). These results suggest a supporting role of miR-130b-3p in the host cells response to *MG* infection.

It is well documented that miRNAs usually exert regulatory effects on gene expressions and cell processes by negatively regulating the downstream target genes at post-transcriptional level. miR-130b-3p has been frequently reported to negatively regulate PTEN by binding PTEN 3′-UTR in mammals [24,41,42,43]. Here, our results also confirmed that PTEN is a direct target of miR-130b-3p in DF-1 cells by bioinformatics analysis, dual luciferase reporter assay, RT-qPCR, and western blot. As one of the most important tumor suppressors, PTEN is a phosphate containing both protein and lipid activities, which plays central roles in cell functions and immune response [44]. Some studies showed that PTEN exerted its inhibitory effect on cell proliferation by blocking cell cycle progression in the G1 phase through down-regulation of cyclins and CDKs proteins, and up-regulation of p21 and p27 proteins, the inhibitors of CDK [45]. Our data indicated that PTEN was significantly down-regulated in both *MG*-infected DF-1 cells (Figure 5b) and *MG*-infected chicken embryo lungs on days of 5, 7, and 9 post-infection (Figure 5a), which was the opposite of that of miR-130b-3p. Since *MG* inhibited PTEN expression and PTEN plays a role in inhibiting cell proliferation and inducing G1 arrest [44,46], we further investigated whether PTEN is the underlying mechanism of miR-130b-3p-mediated cellular protection. Intriguingly, in the gain-of- and loss-of-function experiments, we found that overexpression of miR-130b-3p could significantly decreased the expression of PTEN in DF-1 cells both at mRNA and protein levels (Figure 6a,c), whereas the inhibition of miR-130b-3p led to opposite results (Figure 6b,d). Collectively, these findings suggest that, in response to *MG* infection, up-regulation of miR-130b-3p resulted in inhibition of PTEN expression, thereby promoted *MG*-infected DF-1 cell proliferation by accelerating cell cycle transition from G1 phase to S/G2 phases.

It is now becoming clear that PTEN can negatively regulate the PI3K pathway, and thereby inactivates the PI3K-mediated pathway for cell functions and inflammatory response, leading to a subsequent inactivation of the AKT pathway [33,47]. PI3K, activated by platelet-derived growth factors, has been reported to be essential for inflammatory response to microbial infection and cell damage. Alternatively, down-regulating one of the Class I PI3K members could significantly reduce inflammation severity in models of respiratory disease or allergic inflammation [48,49]. AKT is a serine and threonine kinase activated by PI3K that can promote cell survival by regulating its protein substrates, such as Bad kinase [50] and caspase-9 [51], and can facilitate cell proliferation and cell cycle progression through regulating glycogen synthase kinase-3 (GSK-3) and P27 protein level [52]. Here, our results demonstrated that overexpression of miR-130b-3p resulted in significant increases in the expressions of PI3K (Figure 7a) and AKT (Figure 7b). In contrast, inhibition of miR-130b-3p led to obvious decreases in the expressions of PI3K (Figure 7d) and AKT (Figure 7e). Further support comes from the findings that, in human breast cancer cells, PTEN induced cell growth inhibition, cell death, and G1 arrest by blocking the PI3K/AKT signaling pathway [53]. In lung adenocarcinoma A549 cells, up-regulation of PTEN suppressed cell growth, induced cell cycle arrest, and facilitated cell apoptosis through negatively regulating PI3K/AKT/hTERT pathway [54]. These results strongly supported that PTEN/PI3K/AKT is the underlying molecular mechanism of miR-130b-3p-mediated cellular protection.

Inflammation is one of the major manifestations of the host response to microbial infection or cell damage that hallmarked with release of pro-inflammatory factors, which is regarded as one of the most striking features of CRD [8,37,55]. NF-κB is known to be an important nuclear transcription factor, and plays a predominant role in cell innate immune system [56,57,58]. It has been reported to play central roles in inflammatory response to *mycoplasma* invasion [4,59,60]. For instance, *MG*-derived LAMPs significantly up-regulated IL-1β, IL-6, and IL-8 levels in chicken tracheal epithelial cells through activating the NF-κB pathway [4]. *MG* induced activation of NF-κB leading to up-regulations of IL2, IL6, and TNF-α through the modulation of TLR6 [61]. In quiescent cells, NF-κB is inactivated in the cytoplasm due to the inhibitory protein IκB. Activation of NF-κB depends on the phosphorylation of IκB kinase-α via the PI3K/AKT pathway [62,63]. PI3K/AKT/NF-κB constitutes an important signaling pathway in regulating inflammatory response and cell functions [57,64]. Given the importance of NF-κB in inflammatory response to *MG* and the significant regulatory effect of PI3K and AKT on NF-κB-mediated inflammatory pathway, we further investigate the effect of miR-130b-3p on NF-κB expression. The results showed that the expression of NF-κB in DF-1 cells was strongly correlated with miR-130b-3p. Overexpression of miR-130b-3p led to up-regulation of NF-κB (Figure 7c), whereas inhibition of miR-130b-3p resulted in the opposite result (Figure 7f), suggesting that miR-130b-3p might exert a significant role in regulating NF-κB-mediated inflammatory response. Collectively, it is reasonable to suppose that miR-130b-3p has a strong consequence on *MG*-induced inflammatory response through negatively regulating PTEN/PI3K/AKT pathway.

In conclusion, in response to *MG* infection, the expression of miR-130b-3p was up-regulated in *MG*-infected chicken embryo lungs and DF-1 cells to down-regulate PTEN expression in vivo and in vitro, thereby promoting cell proliferation, cell cycle progression, and inflammatory response through regulating the PI3K/AKT/NF-κB-mediated signaling pathway. These findings help to unveil a novel molecular mechanism of *MG* infection and facilitate the development of research on mechanism of interaction between host and *mycoplasmas*. Additionally, our results support that miR-130b-3p and PTEN might become promising treatment and prevention targets for *MG* infection.

## 4. Materials and Methods

### 4.1. Ethics Statement

In this research, all chicken embryo experiment schemes were conducted in strict accordance with the recommendations provided in the Guide for the Care and Use of Laboratory Animals of the Ministry of Science and Technology of the People’s Republic of China Animal experiments were approved by the Hubei Administrative Committee for Laboratory Animals (Approval No. SYXK-2010-0029, 24 May 2010).

### 4.2. Cell Culture

DF-1 cells, purchased from Huiying (Shanghai, China), were cultured in DMEM (Gibco, Shanghai, China) supplemented with 1% Penicillin-Streptomycin solution (Gibco, Shanghai, China) and 10% fetal bovine serum (Gibco, Shanghai, China) in a carbon dioxide cell incubator with 5% CO_2_ at 37 °C.

### 4.3. Mycoplasma Strains and Growth Conditions

*MG-HS* is a pathogenic strain that was obtained from *MG*-infected chickens in 1998 [13,14]. The *MG-HS* used in this study was deposited and obtained by the State Key Laboratory of Agricultural Microbiology, Huazhong Agricultural University (Wuhan, China). Before use, the strain was cultivated in an improved FM-4 medium with 12% inactivated swine serum and 10% yeast extract until its exponential phase of growth [65]. A color-changing unit experiment (CCU) was applied to examine the concentration of *MG-HS* in medium suspension [66].

### 4.4. Infection Experiments

To set up a *MG* infection model in vitro, we carried out a *MG* infection experiment using DF-1 cells. The experiment including an experimental group and a blank control. Suspension of DF-1 cells were evenly inoculated into 6-well culture dishes and then cultured in medium without antibiotics. When the cells reached 50–60% confluence, the experimental group was treated with 200 μL *MG-HS* (1 × 10^10^ CCU/mL). After 24 h, the total RNA of the cells were extracted by TRNzol Universal according to the reagent kit protocols (TIANGEN, Beijing, China), and stored in −80 °C until further use.

To set up a *MG* infection model in vivo, two-hundred specific pathogen free (SPF) chick embryos (one-day-old) were obtained from Beijing Merial Vital Laboratory Animal Technology Co., Ltd (Beijing, China). SPF chick embryos were hatched in biochemical incubator with temperature of 37.8 ± 0.5 °C and humidity of 55–60%.

At the 9th hatching day, 200 chicken embryos were randomly divided into two groups: infection group and non-infection group. Infection group were treated with 300 µL *MG* (1 × 10^10^ CCU/mL). Non-infection group were treated with the same dosage of diluent as controls. The viability of chicken embryos were examined using an egg candler. 48 h after treatment, dead chick embryos were removed. The mortality rates of chicken embryo in the infection group and non-infection group were 12.3% and 7%, respectively. The whole lung tissue samples from six infected live chicken embryos and six controls were collected on the 9–11th days post-infection, and stored in the RNA safer at −80 °C (BioTeke Co., Ltd., Beijing, China).

### 4.5. DNA Primers and RNA Oligonucleotides

All the DNA primers sequence are presented in Table 1. All the RNA oligonucleotide, including miR-130b-3p mimics (miR-130b-3p), miR-130b-3p mimics negative control (miR-130b-3p-NC), miR-130b-3p inhibitor (miR-130b-3p-Inh) and miR-130b-3p-Inh-NC were synthesized by GenePharm (Shanghai, China) and shown in Table 2.

### 4.6. Reverse Transcription and Quantitative Real-Time (RT-qPCR) Analysis

Total RNA was isolated from infected DF-1 cells and lungs using TRNzol Universal according to the instructions of TRNzol Universal Reagent kit (TIANGEN, Beijing, China). Then, 1 μg of total RNA was inversely transcribed into cDNA using the first strand cDNA systhesis kit (TaKaRa, Tokyo, Japan). Subsequently, the relative expression levels of miR-130b-3p, PTEN, PI3K, AKT, and NF-κB were detected by a real-time qPCR instrument (Bio-Rad, Hercules, CA, USA) with SuperReal PreMix Plus SYBR Green (TIANGEN, Beijing, China). The relative level of miR-130b-3p was normalized to 5S-RNA. The levels of PTEN, PI3K, AKT, and NF-κB were normalized to GAPDH. The total volume of the reaction is 10 μL. Then, the data were statistically calculated using the Ct (2^−ΔΔ*C*t^) method and then analyzed by IBM SPSS Statistics 20. The primers are shown in Table 1. Each experiment group includes three samples. Each sample was measured at least three times independently.

### 4.7. Prediction of miR-130b-3p Target Genes

miRDB (http://www.mirdb.org/) and TargetScan (http://www.targetscan.org/) were applied to predict the candidate targets of miR-130b-3p in chicken. The prediction score, experiment aim, and target genes functions should be taken into consideration to select the appropriate target gene. Qualified prediction score is 60, and the best score is 100. In addition, TargetScan was also used to analyze the conservative of 3′-UTR of miR-130b-3p target in different species (http://www.targetscan.org/). RNAhybrid was applied to predict the RNA secondary structure between miR-130b-3p and the 3′-UTR of its target mRNA, and the minimum free energy (mfe) of RNA secondary structure (https://bibiserv.cebitec.uni-bielefeld.de/rnahybrid/). AmiGO was applied to predict the effect of miR-130b-3p target in chicken (http://amigo.geneontology.org/amigo).

### 4.8. Dual-Luciferase Reporter Assay

A 423bp fragment from PTEN 3′-UTR was ligated to psi-CHECK™-2 vector (Promega, Madison, WI, USA), which was digested by Xho I/Not I in advance, to generate the luciferase reporter vector. The inserted fragment contained the predicted binding site for miR-130b-3p. The primer sequences are presented in Table 1. All amplification products were manifested by DNA sequencing. For the reporter assay, the DF-1 cells in 24-well culture dishes were co-transfected with 10 pmol of miR-130b-3p, miR-130b-3p-NC, miR-130b-3p-Inh, miR-130b-3p-Inh-NC, and 200 ng of the luciferase reporter plasmid using Lipofectamine™ 3000 according to the reagent protocol (Thermo Fisher, Carlsbad, CA, USA). After 48 h, the cells lysates were collected to evaluate the activity of Luc-PTEN 3′-UTR using the automatic microplate reader (Bio-Rad, Hercules, CA, USA) in accordance with the dual luciferase reporter gene detection kit instructions (Promega, Madison, WI, USA). All the Renilla luciferase activities were standardized by firefly luciferase activities. Each experiment group included three duplicates. Each duplicate was measured three times.

### 4.9. Western Blot Analysis

The isolation of total proteins from DF-1 cells was performed 48 h after transfection, utilizing the RIPA lysate with PMSF (100 mM) (Beyotime, Beijing, China). Then, 10 μg total protein were separated with 12% SDS-polyacrylamide gel electrophoresis and transferred into polyvinylidene fluoride (PVDF) membranes. Subsequently, the proteins in membrane were blocked using 5% skimmed milk at 25 °C for 2 h. After that, the under-detected proteins were incubated with the anti-PTEN antibody at 4 °C overnight, followed by washing three times with TBST, and they were finally incubated with secondary antibody for 1 h. After being washed with TBST five times, the protein level of PTEN was evaluated with ECL reagent (Bio-Rad). β-actin served as control. Each experiment group included three duplicates. Each duplicate was measured three times.

### 4.10. Cell Proliferation and Cell Cycle Assay

In line with the protocols, cell proliferation and cell cycle assay were carried out by CCK-8 kit (ZOMANBIO, Beijing, China) and Cell Cycle Detection kit (KeyGEN, Jiangsu, China), respectively. Briefly, DF-1 cells were cultured in 96-well culture dishes until 50–60% confluence. Then, the cells were treated with 10 μL *MG-HS* (1 × 10^10^ CCU/mL) for 2 h. Subsequently, transfection experiments were performed using the infected cells. To avoid *MG* contamination, the cells in the blank group (*MG*-) were grown separately in a sterile incubator. At 24, 48, and 72 h post-transfection, respectively, the absorbance of the cells in each group with CCK-8 solution was scanned at 450 nm by a microplate reader (Bio-Rad) based on the recommended protocols. Each experiment group included three duplicates. Each duplicate was measured three times.

For the cell cycle assay, DF-1 cells were evenly inoculated into 6-well culture dishes. Similarly, cells that had reached 50–60% confluence were incubated with 200 μL of *MG-HS* (1 × 10^10^ CCU/mL). After 2 h, the cells were transfected with the indicated RNA oligonucleotides. 48 h later, the cells grown in 6-well culture dishes were washed with cool PBS, and then fixed in ice-cold 70% ethanol in PBS at 4 °C overnight. According to the instructions, the number of cells distributed in G1, S, and G2 phases were analyzed by a flow cytometer, respectively. Each experiment group included three duplicates. Each duplicate was measured three times.

### 4.11. Statistical Analysis

All the data were statistically analyzed by IBM SPSS Statistics 20 (Armonk, NY, USA). The differences between treatment groups were evaluated by one-way ANOVA with the Duncan’s multiple-range test and Student’s *t*-test. All values are presented as the mean ± SD. Each experiment group included three duplicates. Each duplicate was measured three times. *p* < 0.05 was recognized as statistically significant.

## Figures and Tables

**Table 1:** Sequences of DNA primers.

Name	Primer Sequence (5′-3′)	Accession No.
**Primers for 3′-UTR Cloning**		
PTEN 3′-UTR-F	GAGCAGTAATTCTAGGCGATCGCTCGAGCAATTAGGAACTATAAATATGGCACT	XM-015278701.1
PTEN 3′-UTR-R	AAACGAATTCCCGGGCTCGAGCTGAGCATTACTTTCCATCCC	XM-015278701.1
**Primers for RT-qPCR**		
RT-gga-miR-130b-3p	CTCAACTGGTGTCGTGGAGTCGGCAATTCAGTTGAGTTCAGTTA	MIMAT0001165
gga-miR-130b-3p-F	CTGGTAGGGTACAGTACTGTGATA	MIMAT0001165
gga-miR-130b-3p-R	CTGGTGTCGTGGAGTCGGC	MIMAT0001165
PTEN-F	CCCTTTGAAGACCATAACCCAC	XM-015278701.1
PTEN-R	TTACACCAGTTCGTCCCT	XM-015278701.1
PI3K-F	CTTTTCTGACCCGCTGACTTT	XM-015277626.1
PI3K-R	AATTTCTTACCCACCGCTTC	XM-015277626.1
AKT-F	AAAACAGAGCGACCAAAGCC	NM-205055.1
AKT-R	TGTCTGCTACAGCCTGGATTG	NM-205055.1
NF-κB-F	GCCAGGTTGCCATCGTGT	NM-205129
NF-κB-R	CGTGCGTTTGCGCTTCTC	NM-205129
gga-5s-rRNA-F	CCATACCACCCTGGAAACGC	
gga-5s-rRNA-R	TACTAACCGAGCCCGACCCT	
GAPDH-F	GAGGGTAGTGAAGGCTGCTG	NM-204305
GAPDH-R	CACAACACGGTTGCTGTATC	NM-204305

**Table 2:** Sequences of RNA oligonucleotides.

Name	Sequences (5′–3′)
miR-130b-3p mimics	CAGUGCAAUAAUGAAAGGGCGU
	GCCCUUUCAUUAUUGCACUGUU
miR-130b-3p NC	UUCUCCGAACGUGUCACGUTT
	ACGUGACACGUUCGGAGAATT
miR-130b-3p inhibitor	ACGCCCUUUCAUUAUUGCACUG
miR-130b-3p inhibitor-NC	CAGUACUUUUGUGUAGUACAA
